# The relationship between college students' English learning enjoyment, anxiety, and engagement: the mediating role of grit and the moderating role of flow

**DOI:** 10.3389/fpsyg.2026.1846739

**Published:** 2026-08-04

**Authors:** Xiaofei Zhen, Xiaolian Lian

**Affiliations:** 1 School of Translation and Interpreting Studies, Heilongjiang University, Harbin, Heilongjiang, China; 2 School of Foreign Languages and Literature, Heilongjiang University, Harbin, Heilongjiang, China

**Keywords:** flow, grit, learning anxiety, learning engagement, learning enjoyment

## Abstract

**Background:**

Learning engagement is a key indicator of college English learning quality. In the Chinese EFL context, engagement is closely related to learning emotions, psychological resources, and situational learning states. This study examined learning enjoyment, learning anxiety, grit, flow, and learning engagement within a Control-Value Theory framework.

**Method:**

A cross-sectional survey was conducted with 875 Chinese college students. Standardized questionnaires assessed learning enjoyment, learning anxiety, grit, flow, and learning engagement. Data were analyzed using SPSS 26.0 and AMOS 24.0.

**Results:**

Learning enjoyment was positively associated with learning engagement, whereas learning anxiety was negatively associated with it. Grit was a significant mediator in both associations. Flow moderated the associations between enjoyment and grit, enjoyment and engagement, anxiety and grit, and anxiety and engagement, but not the grit-engagement pathway. At higher levels of flow, the positive associations involving enjoyment were stronger, whereas the negative associations involving anxiety were weaker.

**Conclusion:**

This study extends Control-Value Theory by integrating emotional experiences, trait-like psychological resources, and situational cognitive-affective states. The non-significant flow × grit interaction help clarify the boundary conditions of flow, suggesting that flow may be more relevant to emotion-sensitive pathways than to the stable grit-engagement pathway. The findings suggest that college English teaching should attend to positive emotional experiences, anxiety-related difficulties, grit, and flow-supportive learning conditions.

## 1 Introduction

Learning engagement (LE) is commonly defined as a sustained and positive psychological and behavioral commitment to learning activities, reflected in vigor, dedication, and absorption (Salmela-Aro and Upadaya, 2012). As a key indicator of learning quality, LE is related to their classroom participation, task persistence, strategy use, academic achievement, learning satisfaction, and continuous development (Heilporn et al., 2024; Wong et al., 2023). For college students, English learning is important for academic development, further study, employment, and intercultural communication (Barkaoui, 2025). In Chinese higher education, college English learning is shaped by high-stakes examinations, large class sizes, limited individualized support, and few opportunities for authentic oral communication (Guan, 2026; Zhong et al., 2025). These contextual features may be associated with higher anxiety and lower enjoyment (Lin and Wang, 2025; Liu and Du, 2024). Notably, Lei and Ye (2025) reported considerable variation in students‘ LE during college English learning: some students stay focused and participate actively, whereas others show avoidance, distraction, and shallow LE. Given the key role of LE in college English learning, examining the psychological correlates of students' English LE can contribute to a better understanding of the English learning process and provide more targeted guidance for college English teaching practice.

Control-value theory (CVT) provides a useful theoretical lens for explaining how learning emotions are associated with LE. According to Pekrun (2006), learners' academic emotions are closely related to cognitive, motivational, and behavioral outcomes. In English learning contexts, learning enjoyment can be understood as a positive emotion arising from higher perceived control and task value, which may be associated with greater interest, confidence, and willingness to participate (Lin and Wang, 2025; Liu et al., 2025). In contrast, learning anxiety may arise when learners perceive insufficient control over English learning tasks or feel strong pressure regarding evaluation and failure, which may be associated with reduced concentration, confidence, and sustained participation (Chen and Guo, 2026; Xu and Xie, 2024).

However, associations between learning emotions and LE may not be limited to direct links. Individual psychological resources also need to be considered. Grit may serve as a trait-like resource associated with both learning emotions and LE (Qiao, 2022; Zhao et al., 2024). Enjoyment may be positively associated with learners‘ effort maintenance and interest in English learning, whereas anxiety may be negatively associated with psychological resources and persistence (Jiang, 2025; Younas et al., 2025). On this basis, grit may represent an indirect association between learning emotions and LE. A further consideration is that these associations may vary with learners' situational learning states. Flow, characterized by deep concentration, immersion, and task involvement, may serve as a boundary condition under which the associations of enjoyment and anxiety with grit and LE differ (Liu and Lin, 2025; Wu and Kabilan, 2025). When learners report higher flow, the positive association between enjoyment and learning outcomes may be stronger, whereas the negative association between anxiety and learning outcomes may be weaker.

Existing studies have mainly examined the relationships between learning enjoyment or learning anxiety and LE separately (Chen and Guo, 2026; Lin and Wang, 2025), while other studies have explored the roles of grit or flow in LE in a relatively isolated manner (Fang et al., 2025; Zhao et al., 2024). Although these studies have advanced our understanding of English learning, piecewise approaches leave unclear how learning emotions are related to sustained LE and under what learning-state conditions these relationships vary. In this framework, grit and flow are not treated as interchangeable positive factors. Rather, they are expected to account for different aspects of the emotion-engagement relationship. Grit, as a relatively stable psychological resource, may represent an indirect pathway linking learning emotions with sustained engagement by reflecting learners' persistence, effort maintenance, and long-term goal commitment (Demir, 2024; Zou et al., 2025). Flow, as a situational cognitive-affective state, may capture conditional variation in these associations by indicating when enjoyment and anxiety are more or less strongly related to grit and engagement (Almukhaild and King, 2024; Jia et al., 2025). Thus, examining grit and flow simultaneously help distinguish an indirect association from a boundary-setting association, which cannot be fully clarified by studies examining these constructs separately. To address this gap, the present study extends CVT by incorporating learning enjoyment, learning anxiety, grit, flow, and college students' English LE into an integrated framework. By distinguishing between the indirect role of grit and the boundary-setting function of flow, this study aims to provide a more systematic explanation of college students' English LE in the Chinese EFL context.

## 2 Literature review and hypotheses

### 2.1 Learning enjoyment and learning engagement

Learning enjoyment usually refers to the positive emotional states that learners experience during English learning activities, including interest, pleasure, and a sense of self-actualization (Dewaele and MacIntyre, 2014). According to the CVT, positive academic emotions are closely related to learners‘ perceived control over learning tasks and the value they attach to these tasks, and such emotions are further associated with learning behavior and LE (Pekrun, 2006). Previous studies have generally shown a positive association between English learning enjoyment and LE (Chen et al., 2025; Derakhshan and Fathi, 2024; Liu and Zhou, 2024). For example, Zeng (2021) noted that learners who report greater enjoyment in classroom or self-directed learning tend to use learning strategies more actively, participate more frequently in discussions, and persist in learning tasks (Guo, 2021). Beyond this, learning enjoyment has been associated with learners' motivation, self-efficacy, and positive interpretation of learning feedback, all of which are closely related to LE (Song, 2024; Yang et al., 2026). Accordingly, the following hypothesis was formulated:

H1: College students' English learning enjoyment is significantly and positively correlated with LE.

### 2.2 Learning anxiety and learning engagement

Learning anxiety usually refers to negative emotional states, such as nervousness, uneasiness, or worry, that learners experience during English learning. It may stem from uncertainty about language proficiency, pressure to perform well in class, or concerns about making mistakes (Horwitz, 2001). According to CVT, negative academic emotions are often related to learners' insufficient perceived control over learning tasks or low perceived task value, and these emotions may be associated with lower motivation and behavioral LE (Pekrun, 2006). Previous studies have provided preliminary support for a negative association between learning anxiety and LE (Cao and Han, 2024; Oubibi et al., 2023; Wang et al., 2023). For example, Gullo et al. (2025) found that highly anxious learners may experience distraction of cognitive resources and difficulty maintaining attention during the learning process, which is associated with lower LE. Beyond this, highly anxious learners may avoid challenging tasks, use learning strategies less effectively, and develop stronger negative evaluations of learning difficulties; these tendencies are further related to lower LE (Chen and Guo, 2026). Accordingly, the following hypothesis was formulated:

H2: College students' English learning anxiety is significantly and negatively related to LE.

### 2.3 Learning enjoyment, learning anxiety, grit and learning engagement

Grit refers to a psychological trait that enables individuals to maintain sustained effort and stable interest in the pursuit of long-term learning goals (Duckworth et al., 2007). In learning contexts, learners with grit are usually more able to persevere and remain focused when facing difficulties or frustrations, and they tend to show higher LE in classroom and self-directed learning (Zhao et al., 2024). Gao et al. (2024) found that gritty learners tended to use learning strategies actively, participate in classroom activities, and remain consistently engaged in learning tasks. Qiao (2022) also noted that grit, as a non-cognitive trait, is closely related to students' LE, motivation, and ability to cope with learning challenges. These studies suggest that grit is a key psychological resource for understanding learners' LE.

According to CVT, academic emotions are closely related to learners‘ cognitive, motivational, and behavioral responses (Pekrun, 2006). However, the link between learning emotions and LE may not be limited to immediate emotional reactions. From the perspective of individual psychological resources, grit may serve as a trait-like resource that connects learning emotions with sustained LE. Learning enjoyment may be positively associated with stronger effort maintenance, more stable interest in English learning, and higher grit (Hejazi and Sadoughi, 2023; Jiang, 2025). In contrast, learning anxiety may be negatively associated with psychological resources, persistence, goal commitment, and grit (Pan et al., 2025; Yu et al., 2021). Younas et al. (2025) also suggest that different emotional experiences are closely related to learners' sustained effort and resilience. Taken together, grit may represent an indirect pathway through which English learning enjoyment and anxiety are associated with LE. The following hypotheses are proposed in this study:

H3: Grit functions as a mediator linking college students' English learning enjoyment to LE.
H4: Grit functions as a mediator linking college students' English learning anxiety to LE.

### 2.4 The moderating role of flow

Flow refers to a psychological state of deep concentration, immersion, and task involvement that individuals experience when they are engaged in challenging but manageable activities (Csikszentmihalyi, 1990). In English learning contexts, flow is reflected not only in learners' focused attention and immersion in learning tasks, but also in their positive task experience and sustained participation in the learning process (Almukhaild and King, 2024; Liu and Lin, 2025). When the challenge of a learning task matches learners' abilities, learners tend to report flow and stronger LE (Hui et al., 2025). According to CVT, learning enjoyment and anxiety are closely related to learners' perceived control and task value. Flow may be associated with stronger control-value experiences because it is usually accompanied by clear goals, focused attention, and a sense of involvement in the task (Jia et al., 2025). Under high-flow conditions, positive emotional experiences may be more closely related to persistent effort and active LE (Wu and Kabilan, 2025; Wu and Jiang, 2026). Therefore, the positive associations of learning enjoyment with grit and LE may be stronger when learners experience higher flow. By contrast, when learners are in a high-flow state, their attention is more strongly directed toward the task itself, and the negative associations of anxiety with grit and LE may be weaker (Aubrey et al., 2025; Koçali and Aşik, 2026).

Importantly, the moderating role of flow may be more evident in emotion-related pathways than in the pathway from grit to LE. Although CVT explains how learning emotions are associated with learning outcomes through control and value appraisals (Pekrun, 2006), it does not by itself specify why flow should operate differently across these paths. Learning enjoyment and anxiety are relatively state-sensitive emotional experiences, and their associations with grit and LE may depend strongly on learners' current learning state and attentional resource allocation during learning tasks (Aubrey, 2025; Wang et al., 2024). In contrast, grit reflects a relatively stable tendency toward sustained effort and long-term goal commitment (Demir, 2024; Li, 2025). From a dual-process perspective, learning emotions may operate through relatively immediate affective-attentional processes, whereas grit may rely more on stable, goal-directed self-regulation (Evans and Stanovich, 2013; Wang and Zhang, 2026). When grit is conceptualized as a trait-like resource, its association with LE may depend more on persistence, effort regulation, and long-term goal commitment than on momentary flow experience. Compared with the emotion-related pathways, the moderating role of flow in the association between grit and LE may therefore be weaker or less stable. Based on this reasoning, the following hypotheses and research question are proposed:

H5a: Flow moderates the association between college students' learning enjoyment and LE.
H5b: Flow moderates the association between college students' learning anxiety and LE.
H5c: Flow moderates the association between college students' learning enjoyment and grit.
H5d: Flow moderates the association between college students' learning anxiety and grit.
RQ1: Does flow moderate the association between grit and LE?

**Figure 1:**
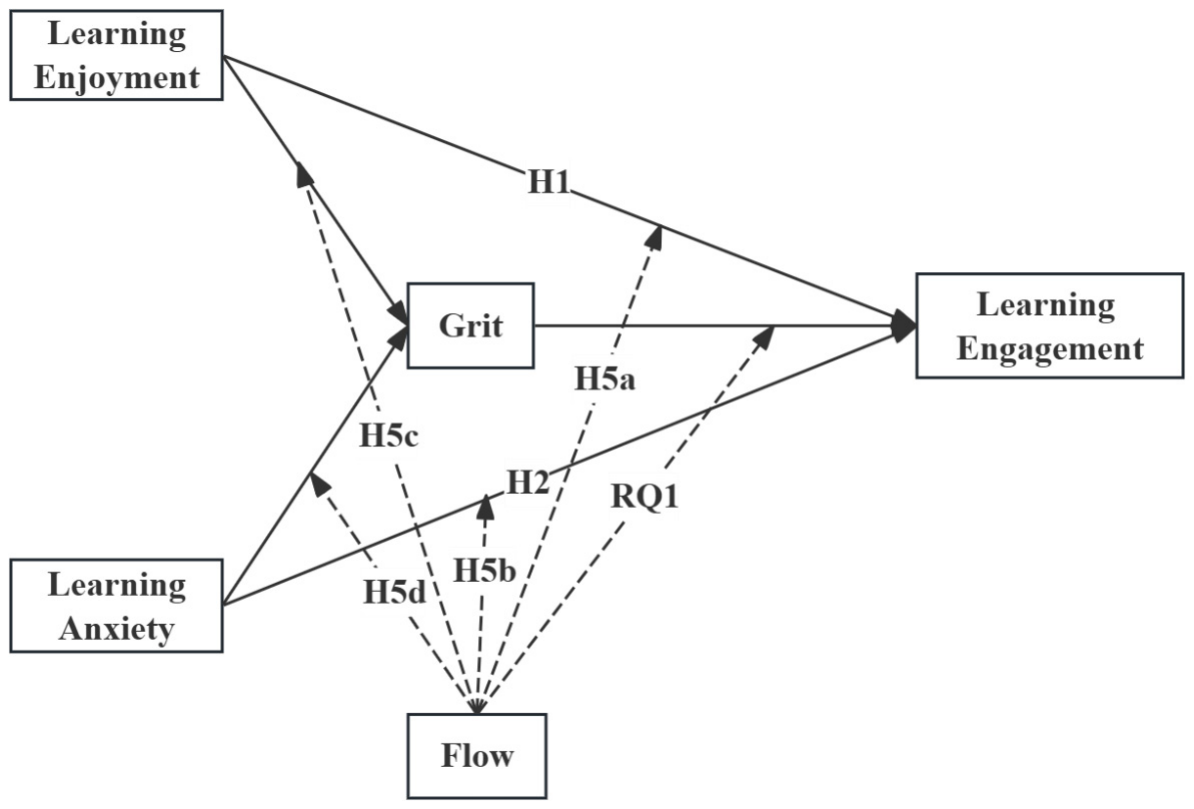
Research hypothesis model.

The theoretical hypothetical model is shown in Figure 1.

## 3 Methods

### 3.1 Sample collection

In this study, a convenience sampling method was used to collect data from college students at four universities in China from February to March 2026: Harbin University, Harbin University of Science and Technology, Harbin Normal University, and Harbin Oriental University. These universities are located in Heilongjiang Province in northern China. With support from the universities, the research team contacted students enrolled in college English courses. In practice, the Registrar's Office helped identify eligible students and distributed an online questionnaire link, together with a brief recruitment notice, to potential participants via the university email system. Before completing the survey, participants read an informed consent form that explained the study goals, procedures, and possible risks. The form emphasized voluntary participation, anonymity, and truthful responses.

A total of 1,000 questionnaires were distributed for this study, and 923 questionnaires were returned, yielding an initial response rate of 92.3%. After excluding invalid responses, including questionnaires with extremely short response times, obvious patterned responses, or extreme response patterns, 875 valid questionnaires were retained for analysis. Among the respondents, 405 were male (46.3%) and 470 were female (53.7%) (Table 1). Because none of the demographic variables showed a significant association with LE in the preliminary analyses, they were not included as covariates in the main model. However, given the potential heterogeneity between English majors and non-English majors, major was further included as a covariate in the robustness analyses.

**Table 1:** Participant demographic information.

Variables	Category	Frequency	Percentage	LE (*M* ±SD)	*t*/F	*p*
Gender	Male	405	46.3%	4.236 ± 1.395	0.577	0.564
	Female	470	53.7%	4.179 ± 1.497		
Age	< 18 years	147	16.8%	4.222 ± 1.454	0.265	0.850
	18–20 years	382	43.7%	4.228 ± 1.422		
	20–22 years	231	26.4%	4.134 ± 1.538		
	>22 years	115	13.1%	4.253 ± 1.369		
Grade	Freshman	281	32.1%	4.214 ± 1.410	0.743	0.527
	Sophomore	167	19.1%	4.323 ± 1.386		
	Junior	172	19.7%	4.089 ± 1.584		
	Senior	255	29.1%	4.196 ± 1.443		
Major	English major	442	50.5%	4.283 ± 1.445	1.611	0.108
	Non-English major	433	49.5%	4.126 ± 1.453		

### 3.2 Instruments

#### 3.2.1 Learning enjoyment

Learning enjoyment was measured using the Chinese version of the foreign language enjoyment scale (CFLES) validated by Li et al. (2018), which has a wide range of applications in the Chinese college student population (Jiang, 2025). CFLES contains 11 items, for example, “I enjoy learning English.” Higher scores indicate greater English learning enjoyment. In this study Cronbach's α = 0.923.

#### 3.2.2 Learning anxiety

Learning anxiety was measured using the English classroom anxiety scale (ECAS) adapted by Liu and Du (2024) from Gardner (1985). The ECAS contains 8 items, for example, “I am always afraid that if I speak in English class, the other students will laugh at me.” Higher scores indicate higher learning anxiety. In this study Cronbach's α = 0.876.

#### 3.2.3 Grit

Grit was measured using the L2 grit scale designed by (Alamer 2021), which has a wide range of applications in the Chinese college student population (Jiang, 2025). The scale contains 12 items, for example, “I will do my best even if it is difficult to learn English.” Higher scores represent higher grit among college students. In this study Cronbach's α = 0.938.

#### 3.2.4 Learning engagement

LE was measured using the learning engagement scale developed by Salmela-Aro and Upadaya (2012), which has a wide range of applications in the Chinese college student population (Shao et al., 2025). The 9-item scale contains three dimensions: vigor, dedication, and absorption. For example, “I feel happy when I am fully engaged in learning English.” Higher scores represent higher LE among college students. In this study Cronbach's α = 0.895.

#### 3.2.5 Flow

Flow was measured using the English learning flow scale (ELFS) developed by Jia et al. (2025). The ELFS consists of 25 items across seven dimensions. For example, “I am totally focused on learning English.” Considering the multidimensional structure of the 25-item English Learning Flow Scale, a higher-order CFA was conducted. The results showed acceptable model fit (χ^2^/ df = 1.392, CFI = 0.992, GFI = 0.968, TLI = 0.991, IFI = 0.992, RMSEA = 0.021, SRMR = 0.017), supporting the use of flow as an overall construct in subsequent analyses. Higher scores indicate greater flow. In this study Cronbach's α = 0.963.

### 3.3 Data analysis

Data were analyzed using SPSS 26.0 and AMOS 24.0. The data analysis followed a sequential procedure. First, descriptive statistics were calculated to summarize the sample characteristics; meanwhile, common method bias (CMB) was examined using Harman's single-factor test and the common latent factor (CLF) method. Second, confirmatory factor analysis (CFA) was conducted in AMOS 24.0 to test the measurement model. The reliability and validity of the measurement model were evaluated using standardized factor loadings, Cronbach's α, composite reliability (CR), average variance extracted (AVE), and discriminant validity. Model fit was assessed using multiple fit indices. Third, after the adequacy of the measurement model was confirmed, structural equation modeling (SEM) was used to test the hypothesized structural model. The significance of the indirect estimates was examined using 5,000 bootstrap resamples and 95% confidence intervals. The final step tested the interaction terms involving flow using the PROCESS macro 4.2 in SPSS. As an additional check, major was included as a covariate in the mediation and moderation analyses.

## 4 Results

### 4.1 Common method bias

Because the variables in this study were measured using participants' self-reported responses, the possibility of CMB was examined. The Harman's single-factor analysis revealed that the first factor without rotation explained 33.267% of the overall variance, remaining below the commonly used 40% threshold (Podsakoff et al., 2003). A complementary CLF analysis was then conducted by re-estimating the measurement model after introducing a common latent factor and comparing its fit with that of the baseline model. The results showed no significant difference between the two models (*p* > 0.05), suggesting that CMB was not severe enough to threaten the validity of the findings.

### 4.2 Measurement model

The measurement model was evaluated in the present Chinese college student sample. Cronbach's α ranged from 0.876 to 0.963, all exceeding the recommended threshold of 0.70 (Nunnally, 1994), indicating satisfactory internal consistency. Standardized factor loadings ranged from 0.553 to 0.821, all above the acceptable threshold of 0.50 (Hair et al., 2019). CR values ranged from 0.902 to 0.966, exceeding the recommended criterion of 0.70 (Hair et al., 2019), indicating good construct reliability. AVE values ranged from 0.533 to 0.599, exceeding the recommended criterion of 0.50 (Fornell and Larcker, 1981; Hair et al., 2019), suggesting adequate convergent validity (Table 2). For discriminant validity, the square root of the AVE for each construct was greater than its correlations with other constructs (Table 3), supporting discriminant validity (Fornell and Larcker, 1981). Overall, these results provide evidence that the measurement tools demonstrated acceptable reliability, convergent validity, and discriminant validity among Chinese college students.

**Table 2:** Reliability and convergent validity.

Variables	Standardized loading	*P*	Cronbach's α	CR	AVE
Enjoyment	0.617-0.814	< 0.001	0.923	0.935	0.569
Anxiety	0.618-0.727	< 0.001	0.876	0.902	0.536
Grit	0.585-0.816	< 0.001	0.938	0.947	0.599
Flow	0.696-0.763	< 0.001	0.963	0.966	0.533
LE	0.553-0.821	< 0.001	0.895	0.915	0.548

**Table 3:** Discriminant validity.

Variables	Anxiety	Enjoyment	Flow	Grit	LE
Anxiety	0.732				
Enjoyment	−0.369	0.755			
Flow	−0.554	0.520	0.730		
Grit	−0.406	0.380	0.408	0.774	
LE	−0.478	0.519	0.468	0.509	0.740

### 4.3 Structural model

The hypothesized structural model was tested using AMOS 24.0. The fit indices indicated that the model fitted the data satisfactorily: CMIN/DF = 1.135, RMSEA = 0.012, GFI = 0.955, AGFI = 0.950, CFI = 0.995, IFI = 0.995, TLI = 0.994, NFI = 0.957, and SRMR = 0.024. As these values were within the recommended ranges, the structural model was considered suitable for subsequent tests of mediation and moderation analyses (Jackson et al., 2009).

### 4.4 Mediation analysis

**Figure 2:**
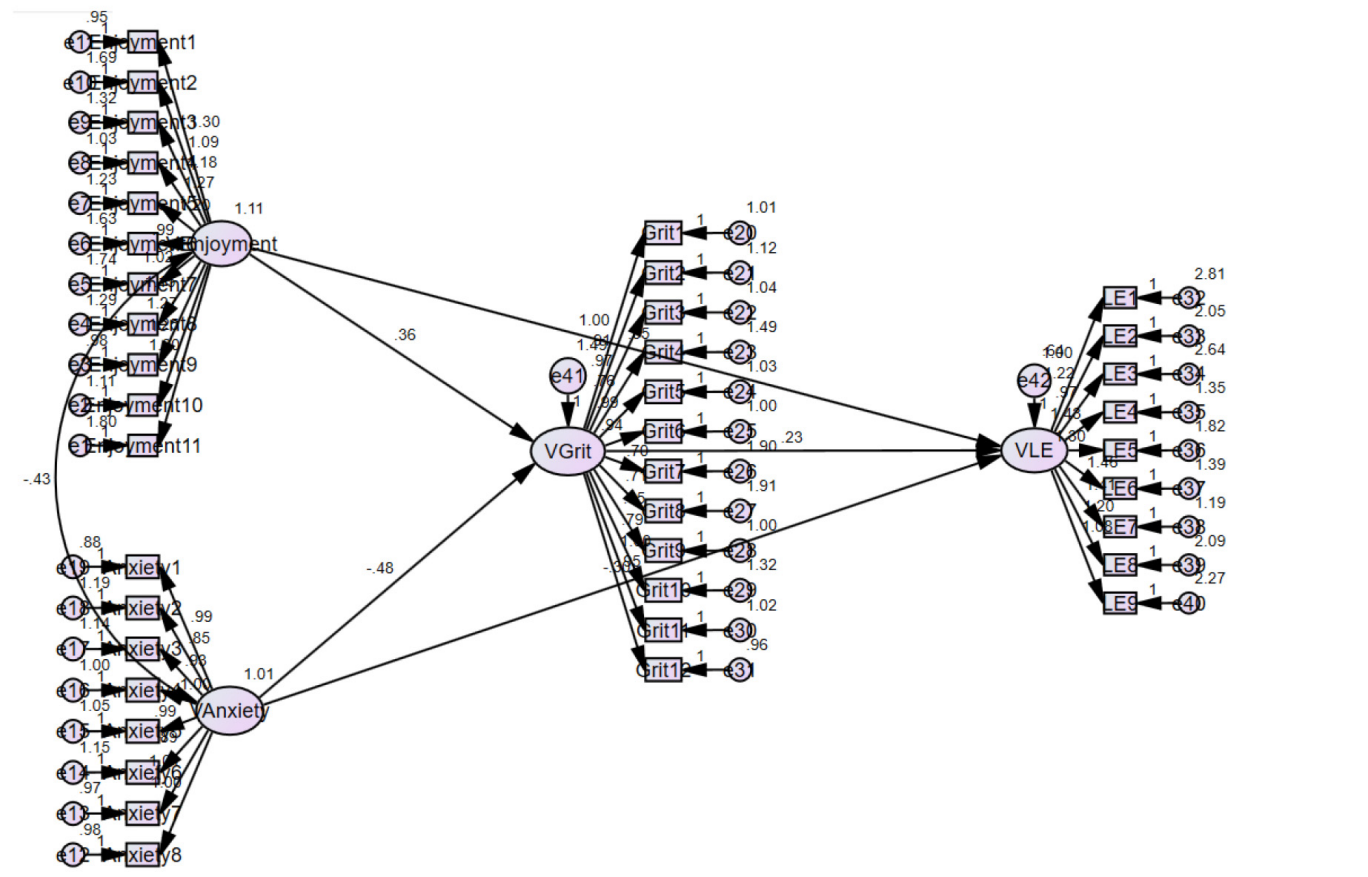
Structural model.

Learning enjoyment was positively associated with LE, with a standardized path coefficient of 0.334, which was consistent with H1. Learning anxiety was negatively associated with LE, with a standardized path coefficient of −0.267, which was consistent with H2. The indirect coefficients linking learning enjoyment and learning anxiety with LE through grit were significant (Table 4 and Figure 2). For the indirect paths, the coefficient for learning enjoyment → grit → LE was 0.078; whereas the coefficient for learning anxiety → grit → LE was−0.098. These results were consistent with H3 and H4.

**Table 4:** Path coefficients of the hypothesized model.

Path relationship	Point estimate	Product of coefficients		Bootstrapping			
				Bias-corrected 95% CI		Percentile 95% CI	
		SE	*T*	Lower	Upper	Lower	Upper
X = Enjoyment, M = Grit, Y = LE							
Enjoyment → LE	0.334	0.042	8.449	0.269	0.397	0.272	0.399
Enjoyment → Grit → LE	0.078	0.014	5.774	0.054	0.107	0.053	0.106
Total effect	0.412	0.032	12.817	0.348	0.474	0.350	0.475
X = Anxiety, M = Grit, Y = LE							
Anxiety → LE	−0.267	0.042	−7.013	−0.340	−0.196	−0.340	−0.196
Anxiety → Grit → LE	−0.098	0.015	−6.512	−0.131	−0.072	−0.129	−0.070
Total effect	−0.366	0.036	−10.248	−0.435	−0.295	−0.435	−0.295

### 4.5 Moderation analysis

**Figure 3:**
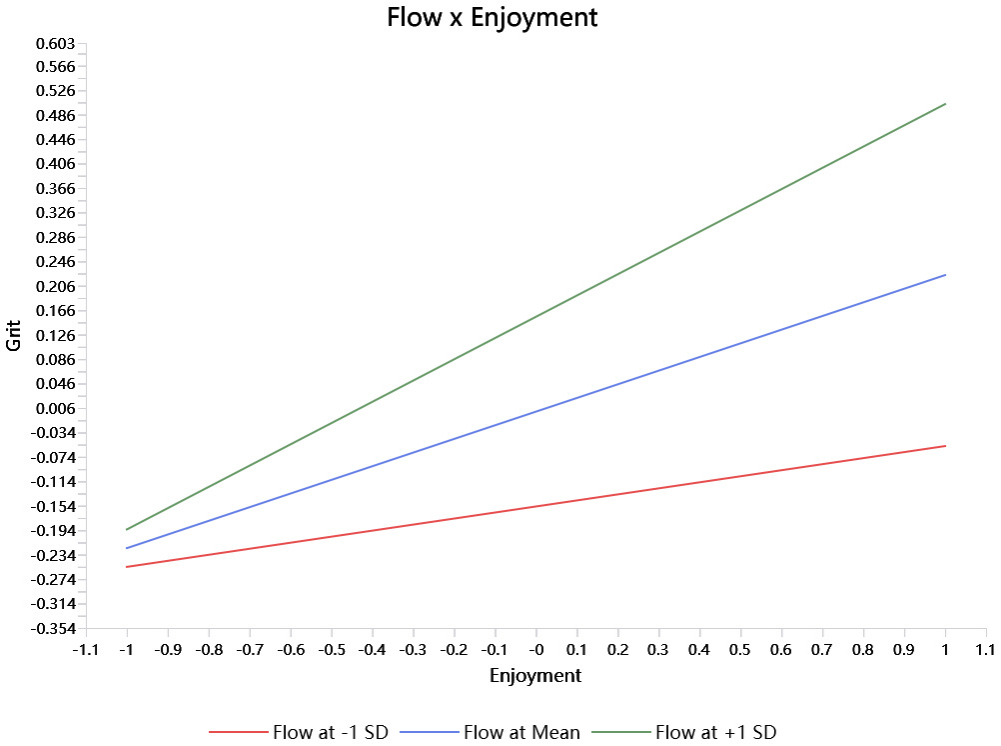
Enjoyment × flow → grit.

**Figure 4:**
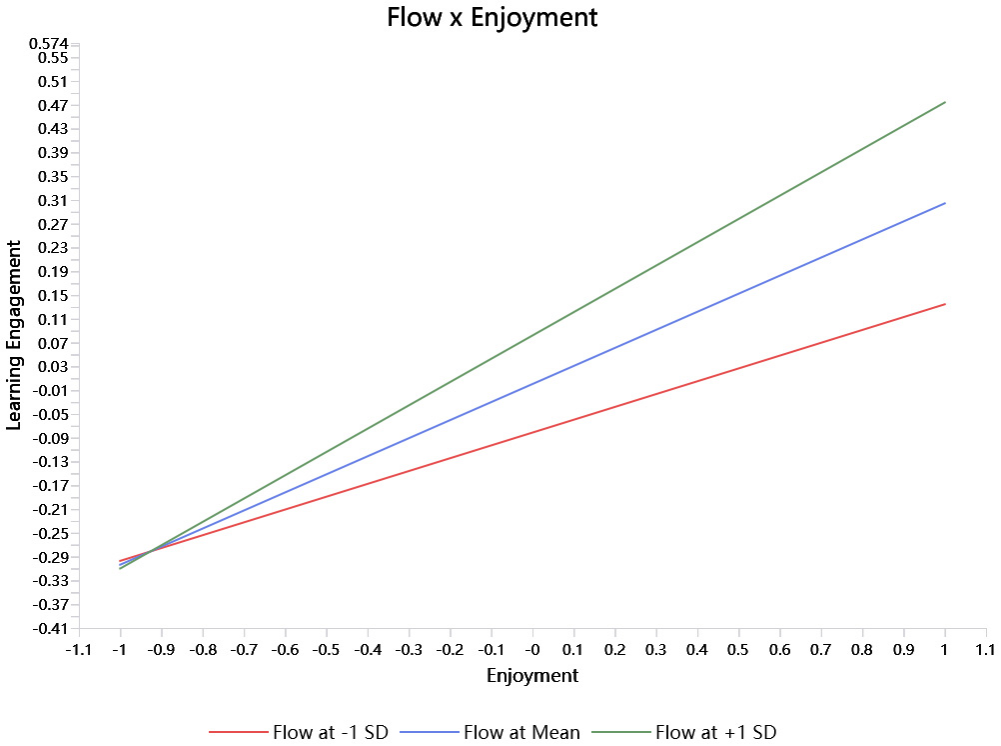
Enjoyment × flow → LE.

**Figure 5:**
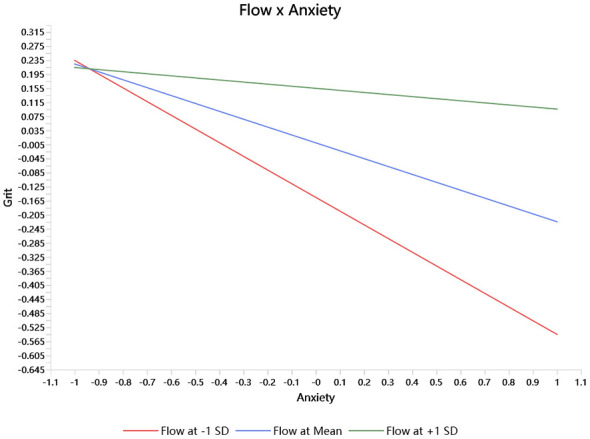
Anxiety × flow → grit.

**Figure 6:**
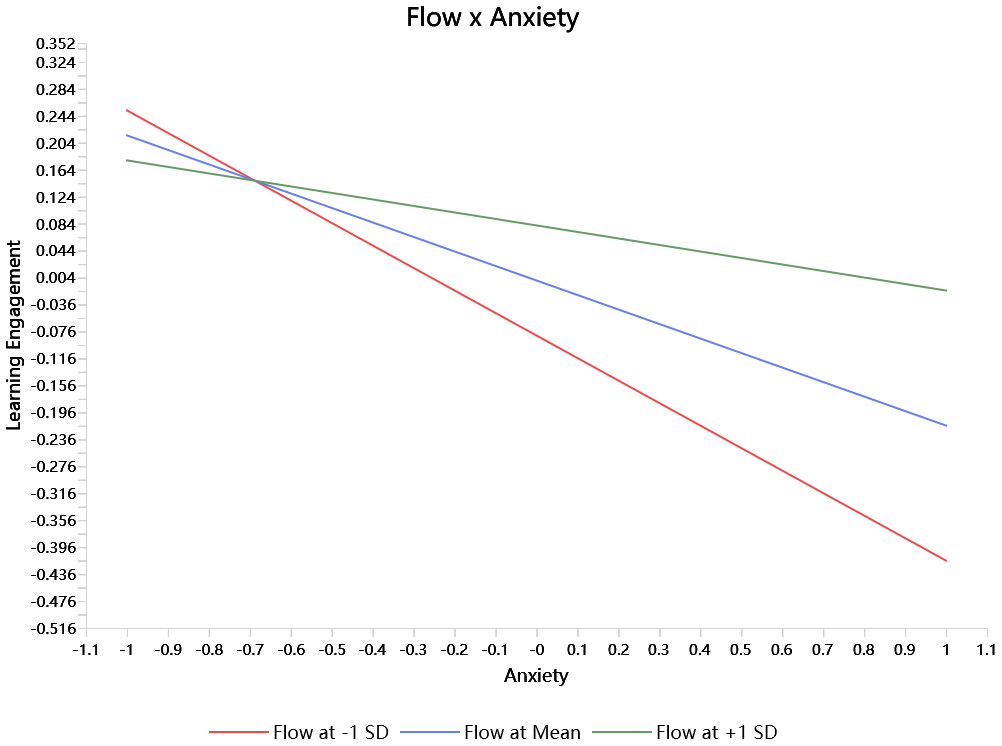
Anxiety × flow → LE.

PROCESS 4.2 was used to examine moderation by flow. With the exception of the Grit × Flow → LE pathway, the interaction terms involving flow were significant in the four emotion-related pathways (Table 5). Simple slope analyses showed that the associations differed across levels of flow in the enjoyment- and anxiety-related pathways. For enjoyment, the slopes were steeper at high flow and flatter at low flow (Figures 3, 4), which was consistent with H5a and H5c. For anxiety, the absolute value of the negative slope was smaller at high flow and larger at low flow (Figures 5, 6), which was consistent with H5b and H5d. These results suggest that flow is a relevant boundary condition for the associations among learning emotions, grit, and LE.

**Table 5:** Moderating analyses of flow.

Path	β	SE	CI (95%)		*p*
			LLCI	ULCI	
Enjoyment × Flow → LE	0.089	0.031	0.028	0.150	0.005
Enjoyment × Flow → Grit	0.066	0.030	0.007	0.126	0.030
Anxiety × Flow → Grit	0.127	0.038	0.053	0.201	0.001
Anxiety × Flow → LE	0.106	0.040	0.027	0.185	0.008
Grit × Flow → LE	0.044	0.029	−0.013	0.101	0.127

### 4.6 Robustness check by major

As a robustness check, major was further included as a covariate in the model to examine whether the findings varied with sample heterogeneity between English majors and non-English majors. The direct paths from learning enjoyment and learning anxiety to LE remained significant after controlling for major, and the indirect associations through grit in the relationships between learning enjoyment, learning anxiety, and LE also remained significant (Table 6). Beyond this, the interaction patterns involving flow in the relationships of learning enjoyment and learning anxiety with grit and LE remained broadly consistent with the original findings (Table 7). Taken together, these results should be interpreted as providing preliminary evidence that the findings were not fully driven by major-related differences.

**Table 6:** Robustness check of the hypothesized model after controlling for major.

Path relationship	Point estimate	Product of coefficients		Bootstrapping			
				Bias-corrected 95% CI		Percentile 95% CI	
		SE	*t*	Lower	Upper	Lower	Upper
X = Enjoyment, M = Grit, Y = LE							
Enjoyment → LE	0.335	0.042	8.468	0.271	0.398	0.273	0.400
Enjoyment → Grit → LE	0.078	0.014	5.571	0.054	0.107	0.054	0.106
Total effect	0.413	0.032	12.906	0.349	0.475	0.350	0.476
X = Anxiety, M = Grit, Y = LE							
Anxiety → LE	−0.264	0.042	−6.938	−0.338	−0.193	−0.337	−0.193
Anxiety → Grit → LE	−0.099	0.015	−6.600	−0.132	−0.073	−0.130	−0.071
Total effect	−0.363	0.036	−10.083	−0.434	−0.292	−0.433	−0.292

**Table 7:** Robustness check of flow interaction terms after controlling for major.

Path	β	SE	CI (95%)		*p*
			LLCI	ULCI	
Enjoyment × Flow → LE	0.091	0.031	0.029	0.152	0.004
Enjoyment × Flow → Grit	0.066	0.030	0.006	0.125	0.030
Anxiety × Flow → Grit	0.126	0.038	0.052	0.201	0.001
Anxiety × Flow → LE	0.107	0.040	0.028	0.186	0.008
Grit × Flow → LE	0.043	0.029	−0.014	0.100	0.137

## 5 Discussion

The purpose of this study was to examine the relationships among learning enjoyment, learning anxiety, grit, flow, and LE among Chinese college students. Drawing on CVT, this study further explored whether grit served as an indirect pathway linking learning emotions with LE and whether flow functioned as a situational boundary condition in these associations. Given the cross-sectional nature of this study, all reported associations should be interpreted as correlational rather than causal. The proposed mediation and moderation paths are theoretically derived and empirically supported, but causal inferences await longitudinal or experimental validation.

First, the results showed that English learning enjoyment was positively associated with LE, whereas English learning anxiety was negatively associated with LE. These associations are consistent with CVT, which suggests that learners' academic emotions are closely related to their perceived control over learning activities and the value they attach to these activities (Pekrun, 2006). In college English learning, enjoyment may reflect learners' higher sense of control, perceived value, and positive task experience, which are closely associated with stronger willingness to participate, strategy use, and persistence in learning tasks (Li et al., 2025). In contrast, anxiety may reflect insufficient perceived control, evaluation pressure, and concern about failure, which are closely related to distraction, lower confidence, and avoidance of challenging learning activities (Chen and Guo, 2026; Pekrun, 2024). Taken together, the opposite associations of enjoyment and anxiety with LE support the relevance of CVT in explaining college students' English LE.

Second, grit significantly mediated the associations of learning enjoyment and learning anxiety with LE. This finding suggests that learning emotions are directly associated with LE and indirectly related to LE through individual psychological resources. According CVT, enjoyment as a positive academic emotion may be associated with learners' stronger effort maintenance and more stable interest in English learning, whereas anxiety may be associated with lower psychological resources and weaker persistence (Bai and Hu, 2025; Jiang, 2025). Grit, as a trait-like psychological resource, may therefore help explain how students with more positive emotional experiences tend to report higher LE, while those with stronger anxiety tend to report lower LE (Arfiandhani and Takeuchi, 2025; Zou et al., 2025). This result extends previous studies by showing that the relationship between learning emotions and LE can be better understood through the indirect pathway of sustained effort and long-term goal commitment.

Third, flow significantly moderated the emotion-related pathways. From the perspective of CVT, flow may indicate the conditions under which academic emotions are more or less strongly associated with learning outcomes. The positive associations of learning enjoyment with grit and LE were stronger at higher levels of flow, whereas the negative associations of learning anxiety with grit and LE were weaker at higher flow. This pattern suggests that flow may function as a situational cognitive-affective state related to variation in the strength of the associations between learning emotions and learning outcomes. When learners report higher flow, their attention may be more strongly directed toward the current English learning task, and their sense of involvement and task control may also be higher (Aubrey et al., 2025). Under such conditions, positive emotional experiences may be more closely associated with sustained effort and active LE (Zhang and Yan, 2025). In contrast, because anxiety is often related to distraction, fear of evaluation, and lower confidence, a high-flow state may correspond to a weaker negative association between anxiety and grit or LE (Liu and Lin, 2025).

Finally, flow did not significantly moderate the association between grit and LE. Rather than treating this null finding as merely non-supportive evidence, it may be theoretically informative because it helps clarify the boundary conditions under which flow operates. Flow appeared to condition the emotion-related pathways, but not the trait-resource pathway from grit to LE. From a dual-process perspective, this pattern is reasonable (Evans and Stanovich, 2013). Emotion processing, including enjoyment and anxiety, may operate partly through relatively fast, automatic, and affect-driven System 1 processes (Evans and Stanovich, 2013; Pekrun, 2006; Wang et al., 2024). These processes are closely tied to immediate attention, task appraisal, and affective experience, and may therefore be more susceptible to situational flow states (Almukhaild and King, 2024; Csikszentmihalyi, 1990). When learners report higher concentration and immersion, the positive association involving enjoyment appears stronger, whereas the negative association involving anxiety appears weaker. By contrast, grit reflects relatively slow, reflective, and goal-directed System 2 processes, including sustained effort, persistence, and long-term goal commitment (Demir, 2024; Duckworth et al., 2007). Once grit functions as a stable self-regulatory resource, its association with LE may depend more on enduring goal commitment and effort regulation than on momentary immersion (Duckworth and Gross, 2014). Taken together, the non-significant Flow × Grit interaction suggests that flow may not operate uniformly across all pathways in the model; instead, its moderating role may be stronger for emotion-sensitive pathways than for trait-resource pathways. This interpretation remains tentative and should be further tested using longitudinal, experience-sampling, or experimental task designs.

### 5.1 Implications

#### 5.1.1 Theoretical implications

This study offers several theoretical contributions. First, it extends CVT in college English learning by moving beyond the direct emotion-engagement association. The findings suggest that learning emotions are directly associated with LE and indirectly associated with LE through grit, a trait-like psychological resource. This provides a more detailed account of how enjoyment and anxiety are linked to sustained LE. Second, this study distinguishes between two mechanisms within the same framework: the indirect pathway of grit and the boundary-setting role of flow. Grit helps account for the indirect association between learning emotions and LE, whereas flow indicates conditions under which these associations become stronger or weaker. By integrating these two mechanisms, this study responds to the limitation of piecewise approaches and provides a more comprehensive account of college students' English LE. Third, the non-significant moderating role of flow in the grit-LE pathway offers a theoretically meaningful boundary condition. Drawing on dual-process theory, the significant flow interaction patterns in the enjoyment- and anxiety-related pathways may reflect the greater sensitivity of fast, automatic, affect-driven System 1 processes to situational learning states. In contrast, grit may represent slower, reflective, goal-directed System 2 processes that rely more on stable self-regulation and long-term commitment than on momentary immersion. This null finding helps delineate where flow is most likely to matter: it appears to be more influential in emotion-sensitive pathways than in the relatively stable grit-engagement pathway. By giving theoretical weight to this null result, the study refines the boundary-setting function of flow and provides a basis for future research to distinguish between situational states and trait-like resources in models of language LE.

#### 5.1.2 Practical implications

The practical implications of this study can be organized as a differentiated instructional framework mapped to the empirical associations. Rather than offering general engagement strategies, the findings suggest that college English teachers should align instructional support with students' emotional experiences, sustained effort, and situational learning states.

First, the finding that learning enjoyment was positively associated with LE, whereas learning anxiety was negatively associated with LE, suggests the need for emotion-oriented instructional support. Teachers should design low-anxiety, interest-driven tasks that help students experience English learning as meaningful and controllable. For students with low enjoyment, teachers may use real-life topics, academic or career-related themes, and choices among task formats such as role plays, debates, short presentations, or video-based responses. For students with high anxiety, teachers can provide lower-pressure conditions through low-risk warm-up tasks, pair or small-group rehearsal before whole-class reporting, anonymous online response tools, clear task requirements, scoring criteria, and model examples before speaking, writing, or presentation activities.

Second, the mediating pattern involving grit indicates that learning emotions are linked to LE partly through students' sustained effort and long-term goal commitment. On this basis, teachers should complement emotion-oriented support with grit-oriented guidance. Long-term English learning goals can be divided into weekly or biweekly milestones, such as completing a vocabulary plan, revising one writing task, recording oral practice, or finishing graded reading. Brief learning logs can also be used to help students record goals, difficulties, strategies, and progress. Teacher feedback should emphasize effort, strategy adjustment, and gradual improvement rather than only final performance.

Third, the moderation results for the emotion-related pathways suggest that flow-oriented task design may be especially relevant when teachers aim to support students with low enjoyment or high anxiety. Teachers can create learning tasks with clear goals, moderate challenge, immediate feedback, and meaningful communication. For example, instead of requiring unprepared speeches, teachers can provide preparation time, sentence frames, peer rehearsal, and clear success criteria. Such flow-supportive conditions may help students keep attention on task completion and limit excessive concern about failure.

Fourth, the non-significant Flow × Grit interaction suggests that flow should not be treated as a universal remedy for all engagement-related mechanisms. Because the association between grit and LE may depend more on stable self-regulation than on momentary flow experience, teachers should not assume that immersive tasks alone are sufficient for gritty students. For these students, practical support should focus more on personal learning plans, progress monitoring, strategy adjustment, and reflection on long-term improvement.

Collectively, these findings suggest a differentiated instructional framework for college English teaching: emotion-oriented support for students with low enjoyment or high anxiety, grit-oriented guidance through milestone setting and reflective monitoring, and flow-oriented task design as a conditional tool rather than a universal remedy. In this framework, teachers first identify the main barrier to students' LE and then provide targeted support that matches the emotional, motivational, or situational mechanism involved.

### 5.2 Limitations and future research directions

The present study still has several limitations. First, this study used a cross-sectional research design. Although it examined the relationships among English learning enjoyment, learning anxiety, grit, flow, and LE, it cannot support rigorous causal inferences about the relationships among these variables. Future research can adopt longitudinal tracking designs, diary studies, or experimental interventions to examine dynamic changes in college students' English learning emotions, grit, and LE across different time points and to test possible temporal or causal mechanisms. Second, the data were derived from self-reports. Although the scales showed good reliability and validity, and the CMB tests indicated no serious common method problem, the findings may still be affected by CMB and social desirability bias. Subsequent studies may combine data from multiple sources, such as teacher evaluations, peer evaluations, classroom behavior observations, or objective indicators such as English grades. Time-separated measurements could also be used to improve the robustness of the findings. Third, although this study further conducted a robustness check based on academic major, it did not collect direct indicators of English proficiency, such as CET scores or course grades. We acknowledge that academic major is an imperfect proxy for English proficiency. Some non-English majors may have high proficiency, and some English majors may have lower proficiency. Therefore, we were unable to directly compare actual proficiency differences between English majors and non-English majors or fully rule out the potential influence of pre-existing proficiency differences on the findings. Future studies should include standardized proficiency measures (e.g., CET-4/6, TOEFL, or course grades) to more precisely test the moderating role of language ability. Finally, the sample mainly consisted of Chinese college students learning English, so the applicability of the findings to learners of other ages, students of different educational types, or different cultural backgrounds remains to be further verified. Future research can conduct comparative studies in different regions, different educational stages, and cross-cultural samples to test the generalizability and contextual applicability of this study's model.

## 6 Conclusion

Based on CVT, this study examined the relationships among English learning emotions, grit, flow, and college students' English LE. Overall, the findings suggest that college students' English LE is not related to a single emotional experience alone, but is associated with emotional factors, individual psychological resources, and situational learning states. Learning enjoyment and learning anxiety showed opposite associations with LE; grit partially mediated these associations, and flow moderated some of the emotion-related paths. These findings suggest that college students' English LE may be closely related to emotional experiences, sustained effort, and immersive learning states. The results provide empirical support for the applicability of CVT in Chinese college English learning contexts. They also suggest that college English teaching should attend to positive emotional experiences, address anxiety-related difficulties, support grit development, and design learning conditions under which flow experiences are more likely to occur.

## Data Availability

The raw data supporting the conclusions of this article will be made available by the authors, without undue reservation.

## References

[B1] Alamer A. (2021). Grit and language learning: construct validation of L2-Grit scale and its relation to later vocabulary knowledge. Educ. Psychol. 41, 544–562. doi: 10.1080/01443410.2020.1867076

[B2] Almukhaild H. King J. (2024). Scaling the apex of engagement: anxious language learners' positive flow experiences during speaking tasks. System 124:103373. doi: 10.1016/j.system.2024.103373

[B3] Arfiandhani P. Takeuchi O. (2025). The interplay of grit, enjoyment, and self-efficacy among Indonesian pre-service EFL teachers: an SEM analysis. Asian-Pac. J. Second Foreign Lang. Educ. 10:23. doi: 10.1186/s40862-025-00330-3

[B4] Aubrey S. (2025). The relationship between task-specific anxiety, enjoyment, and use of planned content across discourse stages of second language learners' spoken task performances. Learn. Individ. Differ. 120:102677. doi: 10.1016/j.lindif.2025.102677

[B5] Aubrey S. Zhang L. Zhou M. R. (2025). Flow in language learning: a systematic review of methodological features. Lang. Teach. Res. doi: 10.1177/13621688251389358. [Epub ahead of print].

[B6] Bai C. Hu J. (2025). The effect of foreign language enjoyment on the willingness to communicate of non-English majors: the mediating role of L2 grit and academic buoyancy. Front. Educ. 10:1614742. doi: 10.3389/feduc.2025.1614742

[B7] Barkaoui K. (2025). The relationship between English language proficiency test scores and academic achievement: a longitudinal study of two tests. Lang. Test. 42, 253–282. doi: 10.1177/02655322251319284

[B8] Cao H.-Q. Han C.-W. (2024). The effect of Chinese vocational college students' perception of feedback on online learning engagement: academic self-efficacy and test anxiety as mediating variables. Front. Psychol. 15:1326746. doi: 10.3389/fpsyg.2024.1326746 38979071 PMC11228330

[B9] Chen C. Guo Z. (2026). From foreign language classroom anxiety to English learning engagement: the roles of cognitive reappraisal and expressive suppression. Front. Psychol. 17:1754113. doi: 10.3389/fpsyg.2026.1754113 41884541 PMC13008866

[B10] Chen J. Mohd Rameli M. R. Cao H. Wang R. (2025). A structural equation model of English learning: exploring the relationship of second language motivation, foreign language enjoyment and learning engagement among Chinese college students. Cogent Educ. 12:2563704. doi: 10.1080/2331186X.2025.2563704

[B11] Csikszentmihalyi M. (1990). Flow: The Psychology of Optimal Experience. New York, NY: Harper & Row.

[B12] Demir Y. (2024). L2 grit: a structured approach to preliminary biblio-systematic review. System 123:103353. doi: 10.1016/j.system.2024.103353

[B13] Derakhshan A. Fathi J. (2024). Grit and foreign language enjoyment as predictors of EFL learners' online engagement: the mediating role of online learning self-efficacy. Asia-Pac. Educ. Res. 33, 759–769. doi: 10.1007/s40299-023-00745-x

[B14] Dewaele J.-M. MacIntyre P. D. (2014). The two faces of Janus? Anxiety and enjoyment in the foreign language classroom. Stud. Second Lang. Learn. Teach. 4, 237–274. doi: 10.14746/ssllt.2014.4.2.5

[B15] Duckworth A. Gross J. J. (2014). Self-control and grit:related but separable determinants of success. Curr. Dir. Psychol. Sci. 23, 319–325. doi: 10.1177/0963721414541462 26855479 PMC4737958

[B16] Duckworth A. L. Peterson C. Matthews M. D. Kelly D. R. (2007). Grit: perseverance and passion for long-term goals. Pers. Soc. Psychol. 92, 1087–1101. doi: 10.1037/0022-3514.92.6.1087 17547490

[B17] Evans J. S. B. T. Stanovich K. E. (2013). Dual-process theories of higher cognition:advancing the debate. Perspect. Psychol. Sci. 8, 223–241. doi: 10.1177/1745691612460685 26172965

[B18] Fang F. Meng Y. Tang L. Cui Y. (2025). The impact of informal digital learning of English (IDLE) on EFL learners' engagement: mediating roles of flow, online self-efficacy, and behavioral intention. Behav. Sci. 15:851. doi: 10.3390/bs15070851 40723634 PMC12292326

[B19] Fornell C. Larcker D. F. (1981). Evaluating structural equation models with unobservable variables and measurement error. J. Mark. Res. 18, 39–50. doi: 10.1177/002224378101800104

[B20] Gao Z. Li X. Liao H. (2024). Teacher support and its impact on ESL student engagement in blended learning: the mediating effects of L2 grit and intended effort. Acta Psychol. 248:104428. doi: 10.1016/j.actpsy.2024.104428 39088995

[B21] Gardner R. C. (1985). Social Psychology and Second Language Learning: The Role of Attitudes and Motivation. London: Edward Arnold.

[B22] Guan Y. (2026). Preferences of Chinese EFL college learners for isolated and integrated form-focused instruction. Discov. Educ. 5:33. doi: 10.1007/s44217-025-01094-1

[B23] Gullo G. Gentile A. Caci B. Alesi M. (2025). Foreign language anxiety and cognition: a systematic review and meta-analysis. Lang. Learn. J. 1–16. doi: 10.1080/09571736.2025.2532516

[B24] Guo Y. (2021). Exploring the dynamic interplay between foreign language enjoyment and learner engagement with regard to EFL achievement and absenteeism: a sequential mixed methods study. Front. Psychol. 12:766058. doi: 10.3389/fpsyg.2021.766058 34721246 PMC8554116

[B25] Hair J. F. Risher J. J. Sarstedt M. Ringle C. M. (2019). When to use and how to report the results of PLS-SEM. Eur. Bus. Rev. 31, 2–24. doi: 10.1108/EBR-11-2018-0203

[B26] Heilporn G. Raynault A. Frenette É. (2024). Student engagement in a higher education course: a multidimensional scale for different course modalities. Soc. Sci. Humanit. Open 9:100794. doi: 10.1016/j.ssaho.2023.100794

[B27] Hejazi S. Y. Sadoughi M. (2023). How does teacher support contribute to learners' grit? The role of learning enjoyment. Innov. Lang. Learn. Teach. 17, 593–606. doi: 10.1080/17501229.2022.2098961

[B28] Horwitz E. (2001). Language anxiety and achievement. Annu. Rev. Appl. Linguist. 21, 112–126. doi: 10.1017/S0267190501000071

[B29] Hui L. Rovers S. Merriënboer J. Donkers J. de Bruin A. (2025). Self-regulation of flow: creating “seemingly effortless” learning among higher education students. Int. J. Educ. Res. 131:102573. doi: 10.1016/j.ijer.2025.102573

[B30] Jackson D. Gillaspy A. Purc-Stephenson R. (2009). Reporting practices in confirmatory factor analysis: an overview and some recommendations. Psychol. Methods 14, 6–23. doi: 10.1037/a0014694 19271845

[B31] Jia F. Meng J. Yu A. (2025). Understanding EFL learners' flow: The development and validation of English learning flow scale. Acta Psychol. 259:105424. doi: 10.1016/j.actpsy.2025.105424 40818422

[B32] Jiang C. (2025). Chinese students' English learning self-efficacy, enjoyment, L2 grit, disciplinary difference, and performance: a moderated mediation model. BMC Psychol. 13:1307. doi: 10.1186/s40359-025-03616-w 41291864 PMC12648954

[B33] Koçali Z. Aşik A. (2026). Exploring mindfulness and language learning: EFL students' journey coping with speaking anxiety. BMC Psychol. 14:370. doi: 10.1186/s40359-026-04161-w 41689143 PMC13005318

[B34] Lei J. Ye W. (2025). Exploring Chinese university students' motivation and engagement in English medium instruction: a latent profiling approach. System 131:103676. doi: 10.1016/j.system.2025.103676

[B35] Li B. Shen W. Ding Y. Pawlak M. Kruk M. (2025). Chinese EFL learners' task control-value appraisals, emotions and behavioral engagement during after-class app-assisted vocabulary learning. Lang. Teach. Res. Q. 48, 216–235. doi: 10.32038/ltrq.2025.48.13

[B36] Li C. Jiang G. Dewaele J.-M. (2018). Understanding chinese high school students' foreign language enjoyment: validation of the Chinese version of the foreign language enjoyment scale. System 76, 183–196. doi: 10.1016/j.system.2018.06.004

[B37] Li G. (2025). Understanding the roles of grit and well-being in L2 achievement among Chinese university english learners. BMC Psychol. 13:576. doi: 10.1186/s40359-025-02933-4 40442786 PMC12121149

[B38] Lin J. Wang Y. (2025). Exploring Chinese university students' foreign language enjoyment, engagement and willingness to communicate in EFL speaking classes. Humanit. Soc. Sci. Commun. 12:650. doi: 10.1057/s41599-025-04948-z

[B39] Liu H. Jiang Y. Nie H. Zhou X. (2025). Unveiling the effects of enjoyment and hope on students' English learning effort. Sci. Rep. 15:26630. doi: 10.1038/s41598-025-11904-z 40696063 PMC12284263

[B40] Liu M. Du N. (2024). A Study of Chinese university students' english learning motivation, anxiety, use of English and English achievement. Sustainability 16:8707. doi: 10.3390/su16198707

[B41] Liu Q. Zhou W. (2024). The impact of teachers‘ emotional support on EFL learners' online learning engagement: the role of enjoyment and boredom. Acta Psychol. 250:104504. doi: 10.1016/j.actpsy.2024.104504 39342900

[B42] Liu X. Lin M. (2025). An empirical study on flow experience regulation in reducing English listening anxiety among high school students. Front. Educ. 10:1618239. doi: 10.3389/feduc.2025.1618239

[B43] Nunnally J. C. (1994). Psychometric Theory, 3rd Edn. New York, NY: McGraw-Hill.

[B44] Oubibi M. Chen G. Fute A. Zhou Y. (2023). The effect of overall parental satisfaction on Chinese students' learning engagement: Role of student anxiety and educational implications. Heliyon 9:e12149. doi: 10.1016/j.heliyon.2022.e12149 36895336 PMC9988470

[B45] Pan Y. Xie C. Wang P. Qian J. Xu L. Zhang Y. . (2025). The relationship between restrained eating and exercise addiction: The chain mediating role of social physique anxiety and grit. Acta Psychol. 260:105520. doi: 10.1016/j.actpsy.2025.105520 40914129

[B46] Pekrun R. (2006). The control-value theory of achievement emotions: assumptions, corollaries, and implications for educational research and practice. Educ. Psychol. Rev. 18, 315–341. doi: 10.1007/s10648-006-9029-9

[B47] Pekrun R. (2024). Control-value theory: from achievement emotion to a general theory of human emotions. Educ. Psychol. Rev. 36:83. doi: 10.1007/s10648-024-09909-7

[B48] Podsakoff P. M. MacKenzie S. B. Lee J.-Y. Podsakoff N. P. (2003). Common method biases in behavioral research: a critical review of the literature and recommended remedies. J. Appl. Psychol. 88, 879–903. doi: 10.1037/0021-9010.88.5.879 14516251

[B49] Qiao R. (2022). A theoretical analysis of approaches to enhance students' grit and academic engagement. Front. Psychol. 13:889509. doi: 10.3389/fpsyg.2022.889509 35859821 PMC9289543

[B50] Salmela-Aro K. Upadaya K. (2012). The schoolwork engagement inventory. Eur. J. Psychol. Assess. 28, 60–67. doi: 10.1027/1015-5759/a000091

[B51] Shao Y. Wu J. Li Y. Lu Q. Wang Z. (2025). The impact of digital technology use on EFL students' English academic performance: the mediating roles of emotional intelligence and learning engagement. BMC Psychol. 13:638. doi: 10.1186/s40359-025-02967-8 40598598 PMC12210690

[B52] Song Y. (2024). Assessing the interactions between learning enjoyment, motivation, burnout, and grit in EFL students: a mixed-methods approach. BMC Psychol. 12:796. doi: 10.1186/s40359-024-02303-6 39741359 PMC11687022

[B53] Wang M. Zhang L. J. (2026). Can grit be perceived and regulated? A mixed-methods exploration of English learners' metacognitive awareness of grit and its impact on achievement and self-efficacy. Lang. Teach. Res. doi: 10.1177/13621688251405049. [Epub ahead of print].

[B54] Wang P. Ganushchak L. Welie C. van Steensel R. (2024). The dynamic nature of emotions in language learning context: theory, method, and analysis. Educ. Psychol. Rev. 36:105. doi: 10.1007/s10648-024-09946-2

[B55] Wang X. Liu Y.-L. Ying B. Lin J. (2023). The effect of learning adaptability on Chinese middle school students' english academic engagement: the chain mediating roles of foreign language anxiety and English learning self-efficacy. Curr. Psychol. 42, 6682–6692. doi: 10.1007/s12144-021-02008-8

[B56] Wong Z. Liem G. A. Chan M. Datu J. A. (2023). Student engagement and its association with academic achievement and subjective wellbeing: a systematic review and meta-analysis. J. Educ. Psychol. 116, 48–75. doi: 10.1037/edu0000833

[B57] Wu W. Kabilan M. K. (2025). Foreign language enjoyment in language learning from a positive psychology perspective: a scoping review. Front. Psychol. 16:1545114. doi: 10.3389/fpsyg.2025.1545114 40391114 PMC12087380

[B58] Wu Y. Jiang L. (2026). EFL learners' flow experience in story continuation writing. System 137:103932. doi: 10.1016/j.system.2025.103932

[B59] Xu Y. Xie Z. (2024). Exploring the predictors of foreign language anxiety: the roles of language proficiency, language exposure, and cognitive control. Front. Psychiatry 15:1492701. doi: 10.3389/fpsyt.2024.1492701 39670145 PMC11634802

[B60] Yang T. Xu W. Li H. T. (2026). Self-efficacy and learning engagement in EFL context: a moderated mediation model of enjoyment, anxiety, and boredom. Acta Psychol. 264:106441. doi: 10.1016/j.actpsy.2026.106441 41679149

[B61] Younas M. El-Dakhs D. A. S. Jiang Y. (2025). Exploring emotional learning and its impact on student behavior, wellbeing, and resilience using structural equation modeling. Sci. Rep. 15:43856. doi: 10.1038/s41598-025-28433-4 41398346 PMC12706065

[B62] Yu Y. Hua L. Feng X. Wang Y. Yu Z. Zi T. . (2021). True grit in learning math: the math anxiety-achievement link is mediated by math-specific grit. Front. Psychol. 12:645793. doi: 10.3389/fpsyg.2021.645793 33889116 PMC8055855

[B63] Zeng Y. (2021). A review of foreign language enjoyment and engagement. Front. Psychol. 12:737613. doi: 10.3389/fpsyg.2021.737613 34456835 PMC8397506

[B64] Zhang J. Yan Y. (2025). The power of grit and enjoyment on engagement of students majoring in teaching Chinese to speakers of other languages (TCSOL) in robot-assisted language learning environment: a self-determination theory perspective. Learn. Motiv. 92:102185. doi: 10.1016/j.lmot.2025.102185

[B65] Zhao H. Zhang Z. Heng S. (2024). Grit and college students' learning engagement: serial mediating effects of mastery goal orientation and cognitive flexibility. Curr. Psychol. 43, 7437–7450. doi: 10.1007/s12144-023-04904-7

[B66] Zhong J. Ismail L. Zheng R. (2025). Improving Chinese EFL learners' oral proficiency through video-enhanced project-based learning: a CAF perspective. Sage Open 15, 1–14. doi: 10.1177/21582440251403309

[B67] Zou M. Azari Noughabi M. Peng C. (2025). Modeling the associations between L2 grit, foreign language enjoyment, and student engagement among Chinese EFL English-major learners: a control-value theory perspective. System 131:103679. doi: 10.1016/j.system.2025.103679

